# An autism-associated serotonin transporter variant disrupts multisensory processing

**DOI:** 10.1038/tp.2017.17

**Published:** 2017-03-21

**Authors:** J K Siemann, C L Muller, C G Forsberg, R D Blakely, J Veenstra-VanderWeele, M T Wallace

**Affiliations:** 1 Neuroscience Program, Vanderbilt University, Nashville, TN, USA; 2 Department of Psychiatry, Vanderbilt University, Nashville, TN, USA; 3 Silvio O. Conte Center for Neuroscience Research, Vanderbilt University, Nashville, TN, USA; 4 Department of Biomedical Science, Charles E. Schmidt College of Medicine, Jupiter, FL, USA; 5 Florida Atlantic University Brain Institute, Florida Atlantic University, Jupiter, FL, USA; 6 Department of Pharmacology, Vanderbilt University, Nashville, TN, USA; 7 Department of Psychiatry, Sackler Institute for Developmental Psychobiology, Columbia University, New York, NY, USA; 8 Center for Autism and The Developing Brain, New York Presbyterian Hospital, New York, NY, USA; 9 New York State Psychiatric Institute, New York, NY, USA; 10 Department of Psychology, Vanderbilt University, Nashville, TN, USA; 11 Department of Hearing and Speech Sciences, Vanderbilt University, Nashville, TN, USA; 12 Kennedy Center for Research on Human Development, Vanderbilt University, Nashville, TN, USA

## Abstract

Altered sensory processing is observed in many children with autism spectrum disorder (ASD), with growing evidence that these impairments extend to the integration of information across the different senses (that is, multisensory function). The serotonin system has an important role in sensory development and function, and alterations of serotonergic signaling have been suggested to have a role in ASD. A gain-of-function coding variant in the serotonin transporter (SERT) associates with sensory aversion in humans, and when expressed in mice produces traits associated with ASD, including disruptions in social and communicative function and repetitive behaviors. The current study set out to test whether these mice also exhibit changes in multisensory function when compared with wild-type (WT) animals on the same genetic background. Mice were trained to respond to auditory and visual stimuli independently before being tested under visual, auditory and paired audiovisual (multisensory) conditions. WT mice exhibited significant gains in response accuracy under audiovisual conditions. In contrast, although the SERT mutant animals learned the auditory and visual tasks comparably to WT littermates, they failed to show behavioral gains under multisensory conditions. We believe these results provide the first behavioral evidence of multisensory deficits in a genetic mouse model related to ASD and implicate the serotonin system in multisensory processing and in the multisensory changes seen in ASD.

## Introduction

Autism spectrum disorder (ASD) is characterized by impairments in social communication as well as the presence of restricted interests and repetitive behaviors.^1, 2^ In addition, sensory abnormalities are highly prevalent in ASD and are now part of the diagnostic criteria.^3, 4, 5^ These changes in response to sensory stimuli have been described in a number of individual sensory systems (for example, vision, touch, hearing), with ongoing research continuing to detail both the specific alterations and their mechanistic bases.^6, 7, 8, 9^ On the basis of the growing evidence for disturbances across multiple sensory systems, there has been an increased focus on examining the integration of information across the different sensory modalities, with a number of studies now detailing impaired multisensory processing in ASD.^10, 11, 12, 13, 14, 15, 16, 17^ The relevance of these multisensory deficits for the autism phenotype is critical, given that multisensory integration has a central role in the construction of coherent perceptual representations, and has been shown to facilitate behavior and perception under a number of circumstances.^18, 19, 20, 21^

The serotonin system has long been implicated in ASD.^22, 23^ Elevated whole-blood serotonin (5-HT), termed hyperserotonemia, is a well-replicated, heritable biomarker present in more than 25% of children with ASD.^24, 25, 26^ Genetic studies in autism also point to the importance of the 5-HT system, including the identification of a group of rare amino-acid-coding variants in the serotonin transporter (SERT) in families with evidence of linkage to the chromosomal region containing SERT.^27^ Each of these variants is known to result in heightened SERT activity.^28, 29^ The most common of these variants, Ala56, is carried by about three million Americans, and in a study of multiplex ASD families, was associated with both sensory alterations and rigid-compulsive behaviors.^27^ The SERT Ala56 knock-in mouse model recapitulates the hyperserotonemia biomarker as well as many of the phenotypic characteristics associated with autism, including abnormalities in social and communicative behaviors as well as repetitive behaviors.^30, 31^

The SERT Ala56 model is of particular interest because it may represent a bridge connecting altered 5-HT function with changes in sensory and multisensory functions in ASD. Numerous animal studies have examined the impact of 5-HT on sensory development ^22, 32, 33, 34^ and processing,^35, 36, 37, 38^ and have demonstrated that 5-HT and SERT are found in a number of sensory brain regions.^22, 33, 39, 40^ Illustrating the importance of 5-HT signaling in these processes, genetic elimination of SERT or the 5-HT-metabolizing enzyme MAOA in the mouse disrupts the development and function of somatosensory cortex.^34, 41, 42, 43^ Similarly, alterations in serotonin function result in abnormal patterns of sensory connectivity.^22, 35, 42, 44^ Furthermore, 5-HT has been shown to modulate neural responses to a variety of sensory stimuli.^38, 45, 46, 47^ Almost all of this work has examined the importance of 5-HT signaling for unisensory (that is, visual alone, auditory alone, somatosensory alone) function.^37, 38, 44, 45, 46^ In contrast, very little is currently known about the role of 5-HT for multisensory function.^36^ As multisensory function augments the integration of information across the different sensory channels, and is thus dependent upon connectivity across brain regions, it may serve as a powerful proxy to probe changes in neural connectivity—changes known to accompany ASD.^48, 49, 50^ Furthermore, sensory and multisensory networks form the foundation for the creation of healthy perceptual and cognitive representations, and thus changes in these sensory networks may scaffold changes in higher-order function.

In an effort to better understand the relationship of the serotonin system to sensory and multisensory function and its potential relevance for autism, here we examined aspects of sensory and multisensory functions in SERT Ala56 mice. Recent work has begun to detail neurophysiological changes in multisensory processing in the mouse,^51, 52, 53^ and we have recently developed a new paradigm to assess behavioral gains under paired audiovisual conditions for, we believe, the first time in the mouse.^54^ Here, we show that SERT Ala56 mice have behavioral deficits in multisensory function that extend beyond changes in unisensory (that is, vision alone and audition alone) performance. These results suggest that abnormalities in the serotonin system may lead to altered multisensory processing in ASD and provide opportunities for further mechanistic studies in rodents and human populations.

## Materials and methods

All animal procedures were in accordance with the National Institutes of Health Guide for the Care and Use of Laboratory Animals and approved by Vanderbilt University. SERT Ala56 knock-in mice were constructed as previously described.^30^ Animals were housed and kept under a food-restricted diet previously outlined.^54^ Eight SERT Ala56 (four male and four female) and eight wild-type (WT) littermate control (four male and four female) mice were used, the sample size was chosen based on our previous study^54^ and experimenters were blinded to the genotypes throughout the behavioral paradigm. SERT Ala56 and WT animals were backcrossed for over 20 generations, maintained on a 129S6/S4 inbred background strain, were the offspring of heterozygous SERT Ala56 parents and were tested at 14 weeks of age.

### Behavioral paradigm

**Figure 1:**
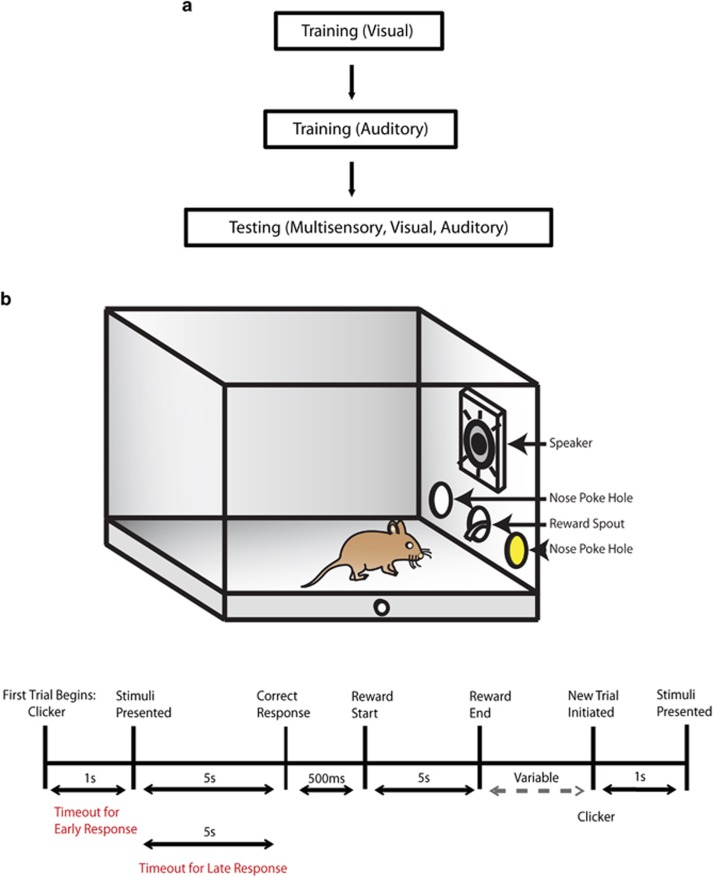
Behavioral task. (**a**) An outlined progression of the behavioral paradigm. (**b**) Above: a diagram of the operant chamber during the presentation of a congruent audiovisual stimulus (represented by the yellow color within the nose poke hole, where the LED was positioned) and by the active speaker. (**b**) Below: a schematic representation of the trial sequence and timing. LED, light-emitting diode.

Animals proceeded through behavioral training and testing procedures previously described (Figure 1).^54^ In this behavioral paradigm, mice were initially trained to respond to visual and auditory stimuli separately for a liquid reward. For unisensory training, mice were first trained to detect and respond to a visual stimulus (that is, an light-emitting diode (LED) that was presented within a nose poke hole on either side of an operant chamber in order to receive a liquid reward. Once mice completed the visual component of the behavioral task for two consecutive days performing at or above a 65% correct criterion, animals then progressed to the auditory-alone component of the task. Under auditory-alone conditions, mice were presented with either white noise or and 8 kHz tone at 85 dB from a centrally located speaker. White noise was associated with a response to the right side of the operant chamber, whereas the tone was associated with a response to the left side of the chamber in order to receive a liquid reward (Figure 1). As previously described,^54^ daily training sessions for these unisensory tasks consisted of 100 trials. After the unisensory training, animals completed testing sessions where visual, auditory and congruent audiovisual (multisensory) pairings were presented. The auditory stimulus was presented simultaneously from a centrally located speaker, with the visual stimulus originating from either the left or right nose poke hole. In addition, all audiovisual trials were congruent (for example, a white noise burst, representing correct responses to the right side, was paired with an LED stimulus in the right nose poke hole). As described in Siemann *et al.*,^54^ mice were tested across stimulus durations ranging from 1 s to 50 ms. The rationale for examining different durations is based on a key principle in the multisensory literature known as inverse effectiveness. This principle states that as the effectiveness (that is loudness, brightness and duration) of the unisensory stimuli decreases, the resultant behavioral gain or benefit increases when these stimuli are combined.^55^ Animals completed 150 trials (50 per sensory modality) that were presented in a pseudorandom order, which lasted up to 120 min per testing session, and animals were tested for 5 days at each of five stimulus durations.

### Data analysis

All behavioral experiments were designed with customized Med-PC IV programs (Med Associates, St. Albans, VT, USA). As previously described,^54^ accuracies measured for visual and auditory training sessions were calculated as percent correct utilizing a 65% correct response rate for two consecutive days. Multisensory gain was calculated as (mean number of correct multisensory trials−mean number of correct best unisensory trials)/(mean number of correct best unisensory trials) × 100. Accuracies were calculated as correct trials/(correct+incorrect trials). Prism 6 (Graphpad Software, La Jolla, CA, USA) was used for all statistical analyses. No significant differences in variance between groups were found. Repeated measures two-way analysis of variances (ANOVAs), Sidak's multiple comparisons tests and s.e.m. were used for all experiments unless otherwise specified.

## Results

### SERT Ala56 mice exhibit comparable behavioral performance to WT mice when trained on visual and auditory stimuli

**Figure 2:**
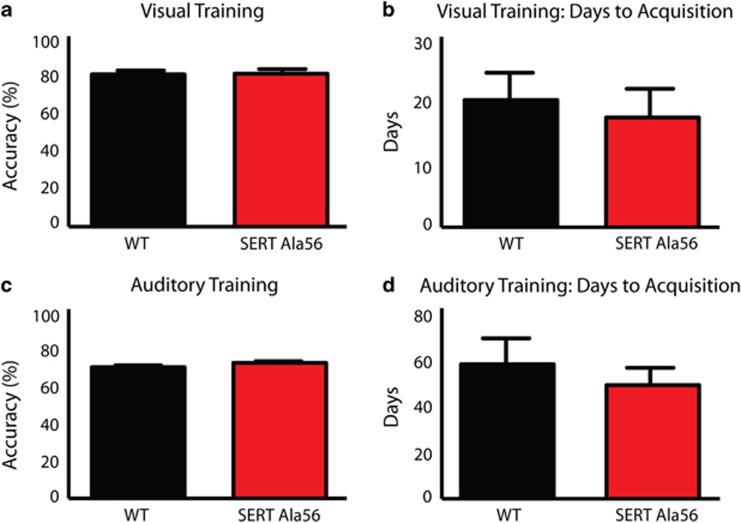
Evaluating behavioral performance for wild-type and SERT Ala56 mice under unisensory training conditions. Unpaired *t-*tests demonstrated no significant differences between genotypes for either (**a**) accuracies (*P*=0.90) or (**b**) days to acquisition (*P*=0.66) under visual training conditions. Wild-type mice completed the visual task after 20.6±4.4 days with a final accuracy of 82.2±2.1%, whereas SERT Ala56 mice completed the visual training in 17.8±4.7 days with a final accuracy of 82.6±2.5%. Unpaired *t*-tests demonstrated no significant differences between wild-type and SERT animals in (**c**) accuracies (*P*=0.21) or (**d**) days to acquisition (*P*=0.52) under auditory training conditions. Wild-type animals completed the auditory training in 58.0±11.1 days and with a final accuracy of 70.8±1.4% and SERT Ala56 mice finished this task after 49.0±7.5 days with a final accuracy of 73.2±1.2%. SERT, serotonin transporter; WT, wild-type.

For the visual task, no significant differences in accuracy (Figure 2a, *P*=0.90) or days to learn (Figure 2b, *P*=0.66) were observed between SERT Ala56 mice and WT littermate controls. Both genotypes took significantly longer to complete auditory training than visual training, but again showed no significant differences between genotypes for accuracies (Figure 2c, *P*=0.21) or days to learn (Figure 2d, *P*=0.52). In summary, no significant differences in behavioral performance were observed between genotypes for learning or performance on the visual and auditory tasks.

### SERT Ala56 mice are less accurate than WT mice under multisensory conditions

**Figure 3:**
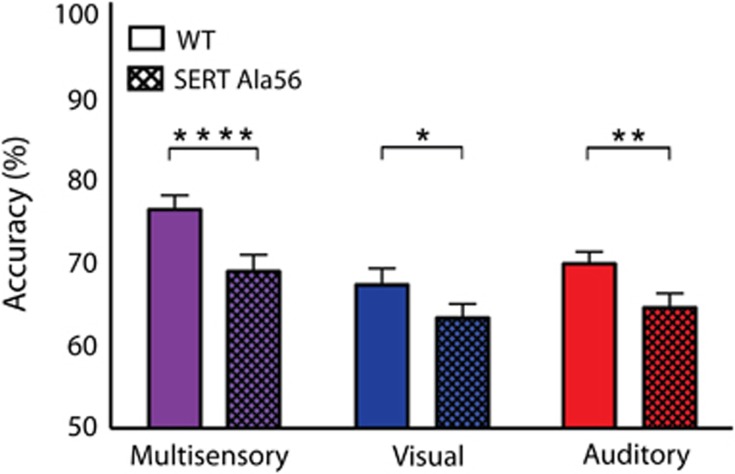
Behavioral accuracies for multisensory, visual and auditory conditions collapsed across stimulus durations. Overall accuracies for these collapsed conditions for wild-type animals were as follows: multisensory—76.4±1.71%, visual—67.3±1.98% and auditory 69.8±1.45%. Accuracies for SERT Ala56 animals were as follows: multisensory—68.9±1.99%, visual—63.3±1.68% and auditory 64.5±1.72%. Significant main effects of genotype (*P*=0.0013; F(1, 39=11.99)) and sensory modality (*P*<0.0001; F(2, 78=51.12)) were observed. Also, significant differences between wild-type and SERT Ala56 animals under multisensory (*P*<0.0001), visual (*P*=0.0215) and auditory conditions (*P*=0.0014) were observed. Behavioral performance was then evaluated within each genotype. For wild-type animals, significant differences between multisensory and visual conditions (*P*<0.0001), multisensory and auditory conditions (*P*<0.0001) and no significant differences between visual and auditory conditions (*P*=0.2000) were found. Similarly for SERT Ala56 mice, significant differences between the multisensory and visual conditions (*P*=0.0007), multisensory and auditory conditions (*P*=0.0093) and no significant differences between visual and auditory conditions (*P*=0.6816) were observed. The significant levels are as follows: (*P<0.05, **P<0.01, ****P<0.0001). SERT, serotonin transporter; WT, wild-type.

After animals completed the auditory training component of the task for two consecutive days performing at or above a 65% correct criterion, mice were tested under conditions in which visual-alone, auditory-alone and paired/congruent audiovisual trials were interleaved. Mice were initially tested on the longest duration condition (1 s) in response to visual, auditory and multisensory stimuli for 5 days. However, in order to modulate the effectiveness of the visual and auditory stimuli in an effort to best assess multisensory gain,^56, 57, 58, 59^ stimulus duration was then varied in intervals of 500, 300, 100 and 50 ms in a blocked design. In an effort to gauge the overarching impact of combined visual–auditory stimulation, we evaluated global behavioral performance by collapsing across stimulus durations. In this analysis, behavioral accuracies under multisensory conditions were significantly greater than those for visual- or auditory-only conditions for both groups (Figure 3). Repeated measures two-way ANOVA revealed significant main effects of genotype (F(1, 39)=11.99; *P*=0.0013) and sensory modality (F(2, 78)=51.12; *P*<0.0001). When evaluating within genotype, both SERT Ala56 mice and WT littermate controls showed significantly improved accuracies under multisensory conditions when compared with both visual and auditory conditions (Figure 3). However, when utilizing Sidak's multiple comparison test and evaluating across genotypes, impaired performance was observed in SERT Ala56 animals in comparison to WT littermate controls under both visual (*P*=0.0215) and auditory conditions (*P*=0.0014). Most strikingly, the most substantial impairment between the genotypes was found under multisensory conditions (*P*<0.0001).

Next multisensory, visual and auditory performances were evaluated across the stimulus durations utilizing repeated measures two-way ANOVAs. When assessing under multisensory conditions, a significant main effect of stimulus duration (F(4, 28)=32.06; *P*<0.0001) and a significant main effect of genotype (F(1, 7)=6.645; *P*=0.0366) were observed (Supplementary Figure 1A). In addition, significant main effects of duration were observed for both unisensory conditions; however, no significant main effects of genotype were found under either visual-only conditions (F(1, 7)=1.819; *P*=0.2194; Supplementary Figure 1B) or auditory-only conditions (F(1, 7)=2.442; *P*=0.1621; Supplementary Figure 1C). Thus, WT animals were significantly more accurate than SERT Ala56 animals under multisensory conditions, and the greatest differences in behavioral performance between genotypes were observed under audiovisual conditions.

When comparing performance based on the sex of the animals irrespective of genotype across stimulus durations, a repeated measures two-way ANOVA demonstrated no significant main effect of sex (F(1, 7)=3.152; *P*=0.1191; Supplementary Figure 2). In addition, the types of errors were measured for each genotype under multisensory conditions. Impaired performance in SERT Ala56 animals was explained by significant differences in the number of incorrect responses (*P*=0.0070), with no significant differences in the number of early (*P*=0.9331) or late responses (0.5016) when compared to WT animals (Supplementary Figure 3). An example of an incorrect response is: if a visual stimulus were presented on the right side congruently with a white noise auditory burst (signifying the animal should respond to the right side to receive a reward) yet the animal responded to the left side of the chamber. These findings demonstrate that WT mice were significantly more accurate under multisensory conditions compared to SERT Ala56 mice, and this was not due to abnormal levels of impulsivity (early errors) or motivation (late errors).

### Multisensory gain is blunted across all stimulus durations in SERT Ala56 mice

**Figure 4:**
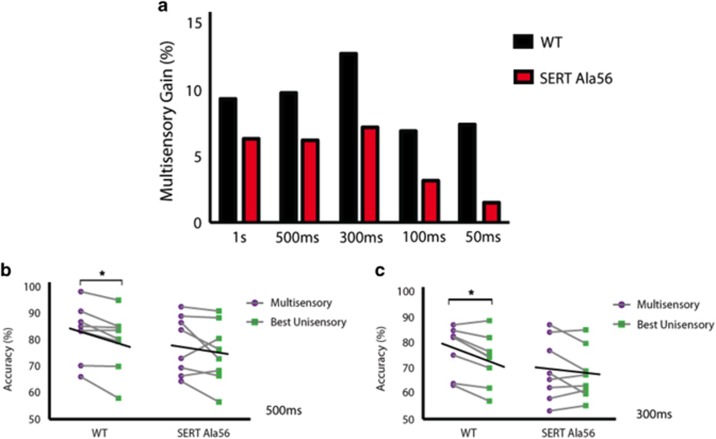
Evaluating multisensory gain across stimulus durations at both the group and individual performance levels. Wild-type animals demonstrated greater multisensory gain than SERT Ala56 animals at the group level at all stimulus durations (**a**). The values for multisensory gain for wild-type mice were as follows: 1 s—9.30%, 500 ms—9.74%, 300 ms—12.70%, 100 ms—6.90% and 50 ms—7.40%. Multisensory gain values for SERT Ala56 mice were as follows: 1 s—6.30%, 500 ms—6.20%, 300 ms—7.20%, 100 ms—3.14% and 50 ms—1.50%. Significant differences in accuracies under multisensory and the best unisensory conditions were observed at both the 500 ms (**b**) and 300 ms (**c**) stimulus durations for wild-type animals. At the 300 ms duration, a repeated measures two-way ANOVA demonstrated a significant main effect of sensory modality (*P*=0.0334; F(1, 7)=6.969) and a significant main effect of genotype (*P*=0.0421; F(1, 7)=6.159; **c**). Significant differences between multisensory and the best unisensory conditions were observed for wild-type mice (*P*=0.02) but not SERT Ala56 mice (*P*=0.36). No significant differences in behavioral accuracies were observed for SERT Ala56 mice for either the 500 ms (**b**) or 300 ms (**c**) stimulus duration. Black lines represent the group average performance under multisensory and the best unisensory conditions. Note the descending slope of these lines, which is apparent for wild-type animals at the 500 and 300 ms durations and is not observed for SERT Ala56 mice. The significant level is: (*P<0.05). ANOVA, analysis of variance; SERT, serotonin transporter; WT, wild-type.

To evaluate the amount of behavioral facilitation resulting from having information available from multiple senses, multisensory gain was calculated using the equation below:


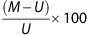


where *M*=average multisensory correct trials and *U*=average best unisensory correct trials.^60^ For WT animals, multisensory gain was found to be significantly different from zero at the 1 s (*P*=0.030), 500 ms (*P*=0.027) and 300 ms (*P*=0.005) conditions. For SERT Ala56 mice, however, there was no statistical evidence of multisensory gain on the group level at any of the stimulus durations. The greatest multisensory gain was seen for both WT and SERT Ala56 mice at the 300 ms duration stimuli, with WT animals exhibiting a greater than 12% gain in behavioral performance. However, SERT Ala56 animals demonstrated a significantly smaller gain in performance (Figure 4a) with this pattern of greater multisensory gain for WT compared to SERT Ala56 animals holding for each of the tested stimulus durations. As this group data could have been due to the superior performance of a few animals, multisensory gain was further evaluated at the level of the individual.

To accomplish this, we compared the behavioral accuracies under multisensory and the best unisensory conditions for each individual mouse at each stimulus duration. First, we utilized correlations between multisensory and best unisensory accuracies to determine whether there was a relationship between these two parameters. When comparing behavioral performance in this manner, significant Pearson correlations were found collapsing across both genotypes (Supplementary Figure 4A), as well as for WT mice (Supplementary Figure 4B) and for SERT Ala56 mice alone (Supplementary Figure 4C). These strong correlations simply highlight that animals, which performed well under multisensory conditions, also had superior performance under the best unisensory conditions, regardless of genotype. Next, we evaluated the individual stimulus durations and found at the 300 ms duration, using a repeated measures two-way ANOVA, a significant main effect of sensory modality (F(1,7)=6.969; *P*=0.0334) and a significant main effect of genotype (F(1, 7)=6.159; *P*=0.0421; Figure 4c). Significant differences between multisensory and the best unisensory conditions were observed for WT mice (*P*=0.02), yet no significant differences were observed for SERT Ala56 mice (*P*=0.36). In addition, at the 500 ms stimulus duration significant differences between multisensory and the best unisensory conditions were found again for WT mice (*P*=0.04, Figure 4b) with no further significant differences observed at the 1 s, 100 or 50 ms durations. Strikingly, no significant differences between multisensory and the best unisensory conditions were observed for SERT Ala56 mice at any of the tested stimulus durations. Therefore, these analyses conducted at the single subject level reinforce the findings from the group data, with both highlighting that multisensory gain is a common feature in WT animals yet absent in SERT Ala56 animals.

## Discussion

This is the first study to demonstrate behavioral changes in multisensory function in a genetic mouse model associated with ASD. SERT Ala56 gain-of-function mutant animals showed no difficulties in learning the respective visual or auditory tasks, but showed significantly diminished multisensory performance in comparison with WT littermate controls. Concordant with what has been demonstrated in a variety of mammalian species,^19, 61, 62^ including recently in the mouse by our group,^54^ behavioral gain in response to paired audiovisual stimulation was found at both the group and individual level for WT mice. In contrast, multisensory gain was substantially influenced (and often eliminated) for the SERT Ala56 animals. In addition, we observed that the principle of inverse effectiveness was impaired in SERT Ala56 animals. This key principle in the multisensory literature states that as the effectiveness of the unisensory components (auditory alone and visual alone) degrade, by decreasing stimulus intensity or shortening stimulus duration, there is a resultant increase in performance when these stimuli are combined (that is, congruent audiovisual presentations).^55^ We previously evaluated this in Siemann *et al.*^54^ and found that WT mice demonstrate this principle as observed in other species.^19, 61^ In the current study we find this principle to be conserved again in WT mice, based on the multisensory gain at the 500 and 300 ms stimulus durations; however, this principle is absent in SERT Ala56 mice based on no significant gain found across the stimulus durations. This impaired behavioral performance manifested as more errors under multisensory conditions for SERT Ala56 animals, suggesting that the finding was not a result of changes in impulsivity (which would manifest as differences in early errors) or motivation (which would manifest as differences in late errors).

Although overall it was demonstrated that multisensory function is atypical in SERT Ala56 animals and that unisensory (that is, auditory alone and visual alone) processing appears to be comparable between the genotypes, it is important to provide a few potential alternate explanations/interpretations and caveats associated with these results. First, the current study does not directly measure visual and auditory acuity, but rather evaluates the ability of animals to utilize information from these individual sensory modalities in order to perform the respective behavioral tasks. Nonetheless, because the animals learned the unisensory components of these tasks in a comparable manner to WT animals, there appear to be no gross differences in unisensory function. However, when collapsing across all stimulus durations, performance differences were found for the three sensory conditions (auditory, visual and audiovisual). Therefore, although the multisensory effects were always greater than those seen within the individual modalities (and were the only significant changes when assessed for a given duration), the audiovisual deficits may reflect an additive or compounding effect of poorer performance under unisensory conditions. These findings then may not necessarily demonstrate a selectivity of multisensory performance deficits; however, the deficits are more severe than what would be predicted based on the unisensory performance of the SERT Ala56 animals. Even if the findings are a result of cumulative effects on auditory and visual functions, they do not weaken the importance of the observed multisensory effects, as these findings illustrate changes in the highly adaptive integration of information across these senses. In addition, it is important to note that the design for the visual and auditory tasks (that is, detection versus discrimination, respectively) may be important differences especially when evaluating multisensory function. Interestingly, recent investigations have begun to assess how these behavioral benefits are conferred under multisensory conditions utilizing different task designs in the mouse model.^63^

Recently, there has been an increased interest in better understanding multisensory processing in individuals with ASD based on the importance of multisensory function for core symptoms such as communication and social interactions.^64, 65^ These human studies have demonstrated atypical multisensory processing in individuals with ASD on both the behavioral and neural levels.^3, 10, 11, 12, 14, 16, 66, 67^ Most germane in the current context, a number of these human studies have detailed weaker multisensory integrative function.^13, 15, 68, 69^ A variety of mouse models associated with ASD have been generated to evaluate the neural underpinnings and associated behaviors.^70^ A recent study demonstrated diminished integration of auditory and somatosensory stimuli in neurons within the insular cortex of multiple mouse lines relevant to autism (BTBR, Shank3, Mecp2 and GAD65).^53^ This work represents the first neural evidence of atypical multisensory responses in mouse models associated with ASD, and demonstrated that a pharmacologic intervention early in development could result in normalizing these atypical multisensory responses.^53^ Even though this study focused on audio-tactile stimulation and our current behavioral study utilized audiovisual stimuli, based on the atypical neural responses to multisensory stimuli, it would be highly relevant to determine whether these mouse models (BTBR, Shank3, Mecp2 and GAD65) demonstrate similar multisensory behavioral deficits as observed in SERT Ala56 mice. To this point, given the absence of any testing for behavioral phenotype in these models, the current study could be a powerful complement to this work and may represent a useful preclinical tool to test therapeutic strategies.

The current findings can be fit within several of the prevailing neurobiologically motivated theories of autism. For example, central coherence is based on the concept that the construction of coherent perceptual representations entails communication across widely distributed brain regions.^71^ In the theory of weak central coherence, individuals with autism are suggested to have impairments in integrating information into more global concepts.^71, 72, 73^ Our current findings in the SERT Ala56 mouse model can be framed in this manner, given that multisensory function augments communication across sensory domains. In addition, these findings could be explained based on an imbalance of excitation/inhibition along with decreased sensory reliability. It has been shown in ASD that there may be an imbalance in the ratio of excitation to inhibition signaling with an increase in excitation compared to inhibition,^74, 75^ which could then result in a less precise or noisier sensory representation.^76, 77^ On the basis of our findings, unisensory function appeared to be comparable across genotypes; however, the most significant deficits were observed once this sensory information was combined under multisensory conditions. One explanation could be that at the cellular level there may be differences in excitatory/inhibitory cell number and distribution even in unisensory along with multisensory brain regions. Therefore, at a circuit level these deficits may then manifest more clearly once multiple sensory channels/connections are combined in multisensory brain regions specifically if there is an atypical cell number or distribution. To further investigate these potential mechanisms, it would be necessary to determine whether the cell distribution in primary sensory cortices as well as higher-order multisensory cortical areas along with the neural connections between these brain regions are atypical in mouse models associated with ASD.

It has been demonstrated that 5-HT can modulate signal-to-noise ratio, receptive field size and structure, and the temporal dynamics of neuronal responses to unisensory stimuli.^38, 47, 78^ For example, one study has demonstrated that serotonin can have an important role in sharpening neural responses to somatosensory stimuli after prolonged periods of visual deprivation in mice^36^—the first evidence illustrating the effects of serotonin on cross-modal plasticity.^36^ Although this study suggests that serotonergic influences may be important in multisensory function, it must be noted that this and other studies have only demonstrated the effects of 5-HT signaling on unisensory function.^37, 38, 47, 78^ Little is known about the role of 5-HT in multisensory function.^36^ Studies have shown serotonergic projections to cortical and subcortical structures to be critical for sensory processing.^22, 32, 39^ The major source of brain 5-HT, the dorsal raphe nucleus, projects to the superior colliculus (SC),^40, 79^ a major hub for multisensory processing^55, 80, 81, 82^ and which expresses multiple 5-HT receptor subtypes.^40, 83, 84, 85^ Furthermore, the SC is likely to have an important role in the behaviors examined in the current study, given its central role in stimulus detection and orientation.^81, 86, 87^ In addition, studies have identified a variety of cortical brain regions important for the processing of multisensory information,^19, 88^ including areas in the rodent model.^51, 52, 53, 89, 90^ One of these regions, area V2L, is of strong interest for the current study, given that it receives direct projections from primary visual and auditory areas^91, 92, 93^ and has been shown to have an important role in multisensory behaviors.^59^ To this point, Hirokawa *et al.*^59^ demonstrated an increase in neuronal activity (that is, increases in cFos staining) in V2L once rats performed a multisensory behavioral task. In regards to our current findings, to more fully elucidate the underlying mechanisms for these multisensory behaviors in both WT and SERT Ala56 mice, future studies may focus on utilizing cFos activity to evaluate neuronal activity in the key structures involved in the assembly of multisensory information including the SC, unisensory cortical regions (that is, V1 and A1) and multisensory cortical areas (that is, V2L). In addition, there is some evidence that the multisensory cortical region in rodents, V2L, projects to the SC.^94, 95^ This is of interest because it suggests that there may be a similar cortical–subcortical circuit that has been previously demonstrated in the cat model system.^96^ Indeed, in this work it has been shown that both the SC^55, 82, 87^ and a cortical multisensory region that projects to the SC, the anterior ectosylvian sulcus,^97, 98^ display robust multisensory integration. Intriguingly, it has been found that the cortical projections to the SC ‘gate' the integrative abilities of SC neurons.^99, 100^ Therefore, based on these foundational observations in the cat and rat model systems, an important next step is the establishment of the circuit mechanisms subserving multisensory processing in the mouse.

While the current study demonstrates atypical behavioral responses under multisensory conditions in SERT Ala56 animals, it is important to note that we have not demonstrated that 5-HT signaling is specifically responsible for these multisensory processing deficits. In order to more fully determine the relationship between multisensory processing and the serotonergic system along with the underlying mechanisms behind these multisensory deficits, future studies may focus on utilizing pharmacology. The SERT Ala56 mouse model allows for the testing of the hypothesis that normalizing serotonin signaling with selective serotonin reuptake inhibitor treatment may reverse or rescue multisensory behavioral deficits observed in these animals. In addition, in order to determine potential developmental versus dynamic changes it would be possible to treat SERT Ala56 animals with selective serotonin reuptake inhibitors at different developmental stages and assess multisensory function. Interestingly, it has previously been shown that multisensory integration can be normalized with pharmacological manipulation of a different neurotransmitter system (GABA) in a mouse model associated with ASD if given early in development as opposed to later in life.^53^ In addition, to further evaluate this relationship it would be possible to chronically treat WT animals with a 5-HT antagonist to limit 5-HT content and mimic the effects observed in SERT Ala56 animals to determine whether this results in similar behavioral deficits under multisensory conditions. These behavioral pharmacology studies in SERT Ala56 animals would more clearly elucidate the potential relationship between 5-HT signaling and multisensory processing and may further elucidate our understanding of the development and mechanistic underpinnings of multisensory function in the context of ASD.

In summary, this is the first study to evaluate and characterize multisensory processing behaviorally in a genetic mouse model relevant to autism. Here, we demonstrate a striking deficit in the ability of mice expressing a hyperfunctional SERT variant to derive behavioral benefits from paired audiovisual stimulation, a result that provides important insights into potential links between serotonergic signaling, multisensory function and autism. We believe that these findings offer great promise as a translational bridge seeking to link genetic, behavioral and neurodevelopmental findings in an effort to better elucidate the contributing role of alterations in sensory function in autism.
