# Supplementary material for: An autism-associated serotonin transporter variant disrupts multisensory processing
Source: Transl Psychiatry. 2017 Mar 21;7(3):e1067–. doi: 10.1038/tp.2017.17 (PMC5416665; doi:10.1038/tp.2017.17)
Supplement: Supplementary Figure Legends [file tp201717x1.docx]

**An Autism-associated Serotonin Transporter Variant Disrupts Multisensory Processing**

Supporting Information

SI Figures

**Supplemental Figure 1.** Evaluating behavioral accuracies under multisensory, visual and auditory conditons across stimulus durations. Under multisensory conditions, a repeated measures 2-way ANOVA demonstrated a significant main effect of stimulus duration (p <0.0001; F(4, 28) = 32.06) and a significant main effect of genotype (p = 0.0366; F(1, 7) = 6.645) but no significant interaction effect (p = 0.6590; F(4, 28) = 0.6097) was observed (A). Under visual conditions a main effect of stimulus duration was observed (p <0.0001; F(4, 28) = 31.92), yet no significant main effect of genotype (p = 0.2194; F(1, 7) = 1.819) nor a significant interaction effect (p = 0.9613; F(4, 28) = 0.1503) were found (B). These findings were similar under auditory conditions, with a main effect of stimulus duration (p <0.0001; F(4, 28) = 24.84) being observed, but no significant main effect of genotype (p = 0.1621; F(1, 7) = 2.442) nor a significant interaction effect (p = 0.7012; F(4, 28) = 0.5491) were found (C). The significant levels are as follows: (* = p < 0.05, ** = p < 0.01).

**Supplemental Figure 2.** Measuring effects of sex on behavioral accuracies under multisensory conditions. When comparing across sex, a repeated measures two-way ANOVA demonstrated a significant main effect of stimulus duration (F(4, 28) = 26.30; p < 0.0001) yet no significant main effect of sex (F(1, 7) = 3.152; p = 0.1191) nor a significant interaction effect (F(4, 28) = 1.028, p = 0.4103) were observed.

**Supplemental Figure 3.** Evaluating the types of errors made under multisensory conditions between wild type and SERT Ala56 mice. Significant main effects of error type (p = 0.0003), genotype (p = 0.0003) and a significant error type x genotype interaction effect (p = 0.0475) were observed. The significant level is: (* = p < 0.05).

**Supplemental Figure 4.** Determining the relationship between accuracies under multisensory and the best unisensory conditions. Signficant Pearson correlations between accuracies under multisensory and the best unisensory conditions were found at every stimulus duration - 1s: (r = 0.7017, p < 0.0001), 500ms: (r = 0.8073, p < 0.0001), 300ms: (r = 0.8554, p < 0.0001), 100ms: (r = 0.7759, p < 0.0001) and 50ms: (r = 0.5414, p = 0.0012) when performance was collapsed for all mice (A). For wild type mice the values were observed as: 1s: (r = 0.7660, p = 0.0044), 500ms: (r = 0.9228, p = 0.0001), 300ms: (r = 0.8980, p = 0.0003), 100ms: (r = 0.8401, p = 0.0014) and 50ms: (r = 0.4013, p = 0.0917) (B). For SERT Ala56 mice the values were: 1s: (r = 0.7714, p = 0.0250), 500ms: (r = 0.6937, p = 0.0103), 300ms: (r = 0.8382, p = 0.0014), 100ms: (r = 0.6712, p = 0.0128) and 50ms: (r = 0.3733, p = 0.1076) (C).
