# Supplementary material for: An autism-associated serotonin transporter variant disrupts multisensory processing
Source: Transl Psychiatry. 2017 Mar 21;7(3):e1067–. doi: 10.1038/tp.2017.17 (PMC5416665; doi:10.1038/tp.2017.17)
Supplement: Supplementary Figure 1 [file tp201717x2.pdf]

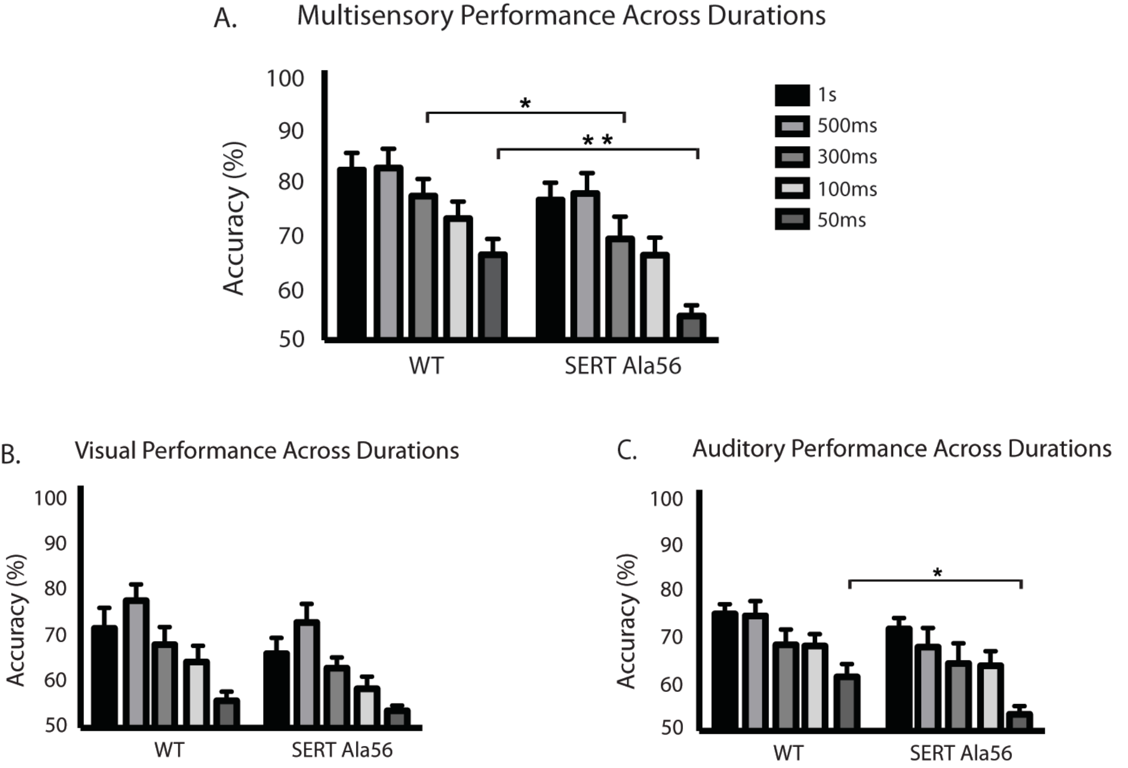
A.
Multisensory Performance Across Durations
Accuracy (%)
100
90
80
70
60
50
*
* *
1s
500ms
300ms
100ms
50ms
WT
SERT Ala56
B.
Visual Performance Across Durations
Accuracy (%)
100
90
80
70
60
50
WT
SERT Ala56
C.
Auditory Performance Across Durations
Accuracy (%)
100
90
80
70
60
50
*
WT
SERT Ala56
