# Supplementary figures and images for: An autism-associated serotonin transporter variant disrupts multisensory processing
Source: Transl Psychiatry. 2017 Mar 21;7(3):e1067–. doi: 10.1038/tp.2017.17 (PMC5416665; doi:10.1038/tp.2017.17)

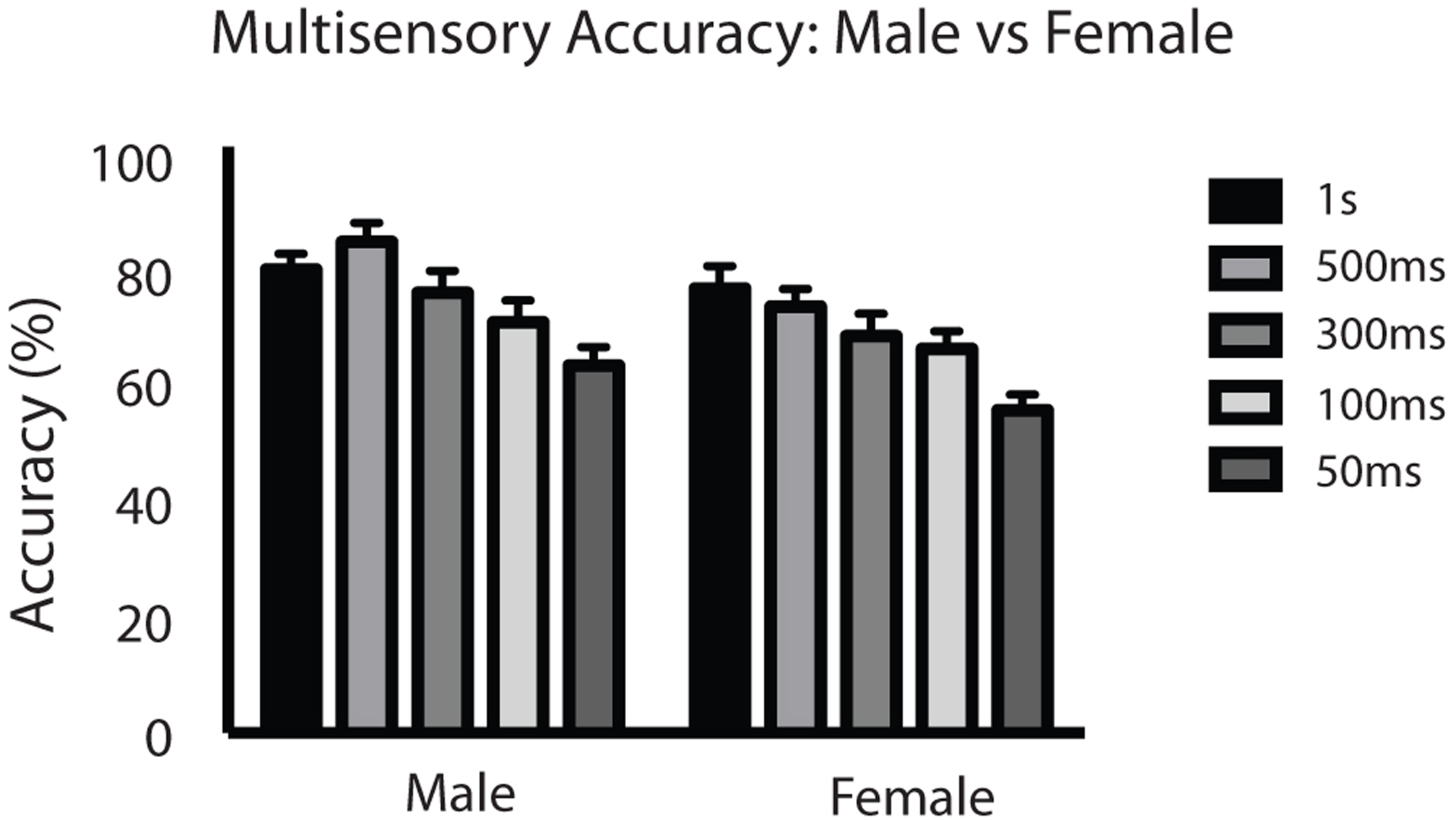

Supplement: Supplementary Figure 2 [file tp201717x3.tif]

Number of Errors

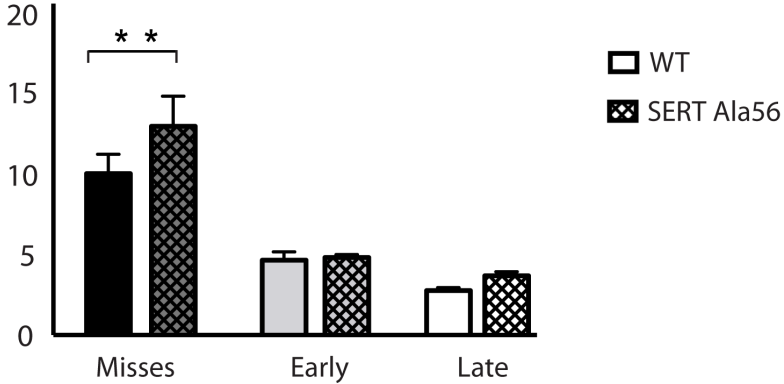

Supplement: Supplementary Figure 3 [file tp201717x4.pdf]

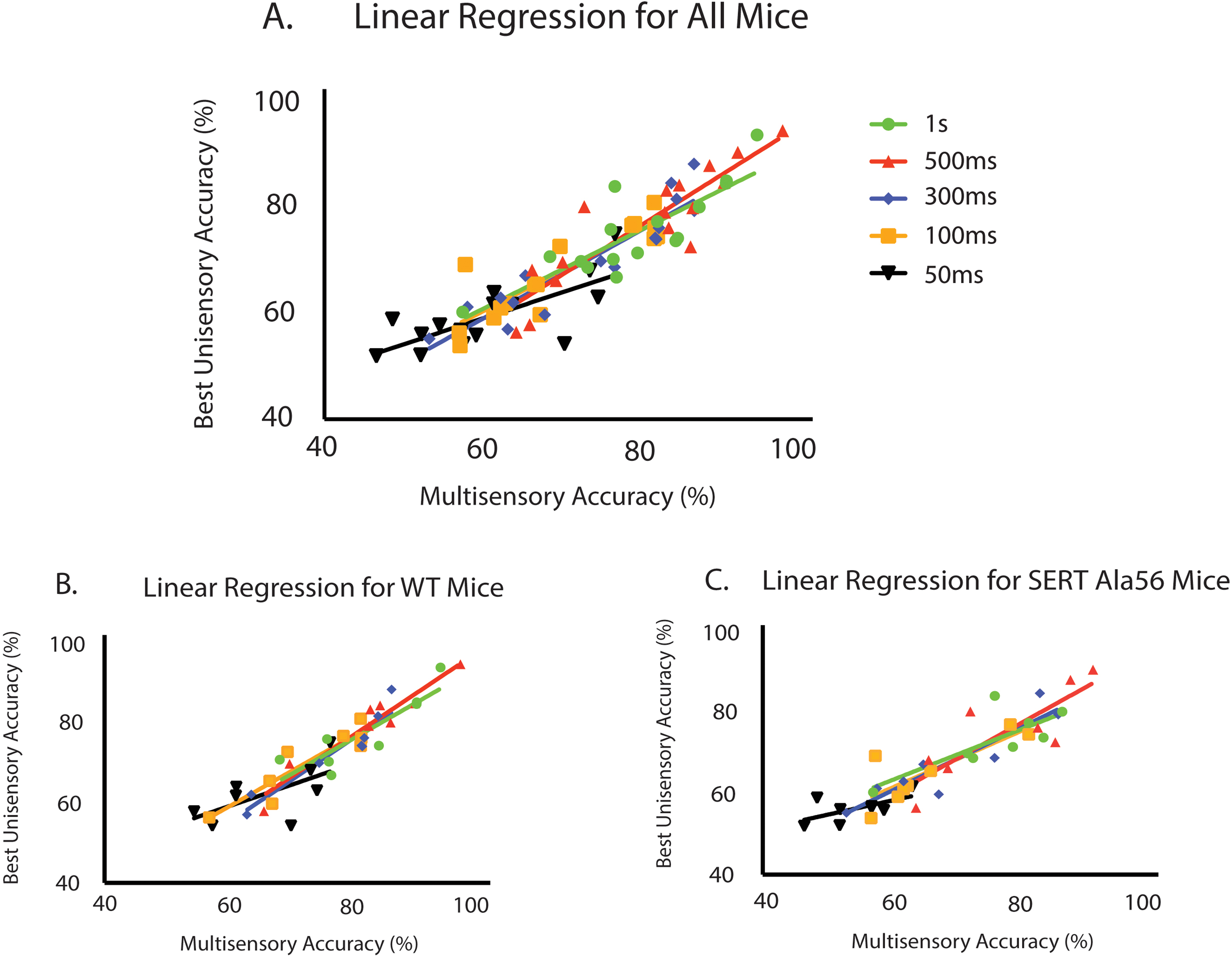

Supplement: Supplementary Figure 4 [file tp201717x5.tif]
